# Photon-upconverting chiral liquid crystal: significantly amplified upconverted circularly polarized luminescence†
†Electronic supplementary information (ESI) available. See DOI: 10.1039/c8sc03806f


**DOI:** 10.1039/c8sc03806f

**Published:** 2018-10-02

**Authors:** Xuefeng Yang, Jianlei Han, Yafei Wang, Pengfei Duan

**Affiliations:** a College of Chemistry , Key Lab of Environment-Friendly Chemistry and Application of the Ministry of Education , Xiangtan University , Xiangtan 411105 , P. R. China; b CAS Center for Excellence in Nanoscience , CAS Key Laboratory of Nanosystem and Hierarchical Fabrication , Division of Nanophotonics , National Center for Nanoscience and Technology (NCNST) , No. 11 ZhongGuanCun BeiYiTiao , Beijing 100190 , P. R. China . Email: duanpf@nanoctr.cn; c Science and Engineering , Jiangsu Collaboration Innovation Center of Photovoltaic Science and Engineering , Changzhou University , Changzhou 213164 , P. R. China; d University of Chinese Academy of Sciences , Beijing 10049 , P. R. China

## Abstract

In this work, we demonstrate a room-temperature chiral liquid crystal which shows remarkable photon upconverted circularly polarized luminescence (UC-CPL). Circularly polarized luminescent materials with higher dissymmetry factor (*g*_lum_) underpin the basis for future applications. Since most chiral organic molecules have only a small *g*_lum_, it is vital to explore a new pathway to amplify the *g*_lum_ of organic compounds. Here, by dispersing a chiral emitter and a triplet donor into a room-temperature nematic liquid crystal, highly efficient triplet–triplet annihilation-based photon upconversion (TTA-UC) and UC-CPL were observed in the induced chiral nematic liquid crystal (N*LC). Moreover, this system could simultaneously amplify the promoted circularly polarized luminescence (CPL) and the upconverted circularly polarized luminescence. The dissymmetry factors *g*_lum_ of CPL and UC-CPL have been amplified by three orders of magnitude and one order of magnitude, respectively.

## Abstract 2

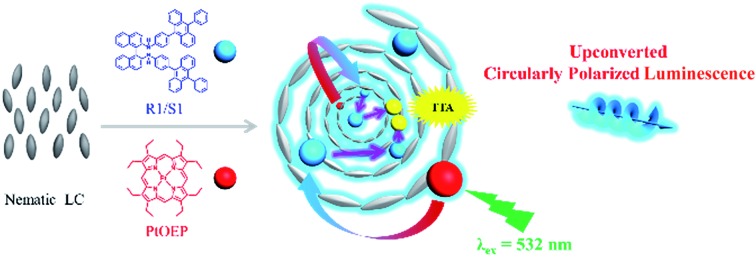
By blending a chiral acceptor and a sensitizer into a nematic liquid crystal, a chiral nematic liquid crystal showing amplified upconverted circularly polarized luminescence could be obtained.

## Introduction

Photon upconversion, the process of converting low energy photons into high energy photons is interesting, both from a fundamental and application perspective. Particularly, due to the wide potential applications ranging from energy to biology, triplet–triplet annihilation-based photon upconversion (TTA-UC, Fig. S1†) has attracted attention in recent years. To date, TTA-UC has been demonstrated extensively in various matrices including dilute solutions, non-volatile liquids, gels and polymers.^1^–^15^ However, the emission behaviours in liquid crystals were rarely reported. Researchers have tried to tune the emission direction or the stimuli-responsiveness of TTA-UC through liquid crystal matrices.^16^–^19^ The chiroptical properties, *e.g.* circularly polarized luminescence (CPL), of TTA-UC in liquid crystals have never been explored.

Circularly polarized luminescence has been attracting increasing attention^20^–^32^ because of its remarkable advantages including its extensive optical information and lack of angular dependence, which has been widely used for various applications.^33^–^38^ Ordinarily, luminescence dissymmetry factor *g*_lum_ is used to evaluate the level of CPL, whose value is in the range of –2 to 2, and is defined as 2(*I*_L_ – *I*_R_)/(*I*_L_ + *I*_R_), where *I*_L_ and *I*_R_ are the intensities of the left- and right-handed circularly polarized emissions, respectively. Generally, for pure organic chiral molecules, *g*_lum_ is extremely small within the range of 10^–5^ to 10^–3^. Thus, exploring new pathways to amplify the *g*_lum_ of organic compounds is a vital issue in developing chiroptical materials. Kawai and co-workers had demonstrated that supramolecular self-assembly could remarkably enhance the *g*_lum_ in chiral perylene bisimide systems.^39^,^40^ Tang and co-workers had amplified *g*_lum_ by introducing the concept of aggregation-induced emission (AIE).^41^ Very recently, we have found that energy transfer in a hybrid system could significantly amplify the *g*_lum_ value, including Förster resonance energy transfer (FRET) and TTA-UC process.^42^,^43^ However, an effective method to amplify the *g*_lum_ value of upconverted circularly polarized luminescence (UC-CPL) has never been reported.

**Fig. 1:**
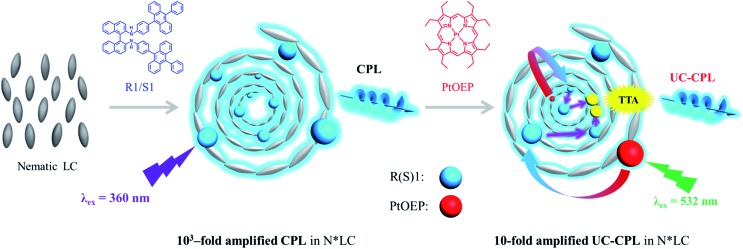
N*LC could be obtained by doping chiral acceptor R(S)1 into the nematic liquid crystal, which could emit circularly polarized light by excitation with a 360 nm light. The dissymmetry factor *g*_lum_ was amplified by three orders of magnitude compared with the diluted solution. Subsequently both upconverted emission and upconverted circularly polarized emission could be observed sensitized by PtOEP. The *g*_lum_ value of UC-CPL was amplified by one order of magnitude compared with the diluted solution.

In this work, we show the first example of enhanced UC-CPL emission in a chiral liquid crystal (N*LC). N*LC could be easily obtained by blending a chiral emitter R(S)1 (Fig. 1) with an achiral nematic liquid crystal, whose *g*_lum_ value was three orders of magnitude higher compared with the dilute solution sample. Subsequently, by mixing with an appropriate amount of sensitizer, Pt(ii)octaethyl porphyrin (PtOEP), both upconversion emission and upconverted circularly polarized luminescence (UC-CPL) could be observed. More importantly, compared with the UC-CPL in dilute solution, the *g*_lum_ value of UC-CPL in the N*LC was significantly amplified by one-order of magnitude (Fig. 1).

## Results and discussion

### Upconversion behavior in a chiral liquid crystal

**Fig. 2:**
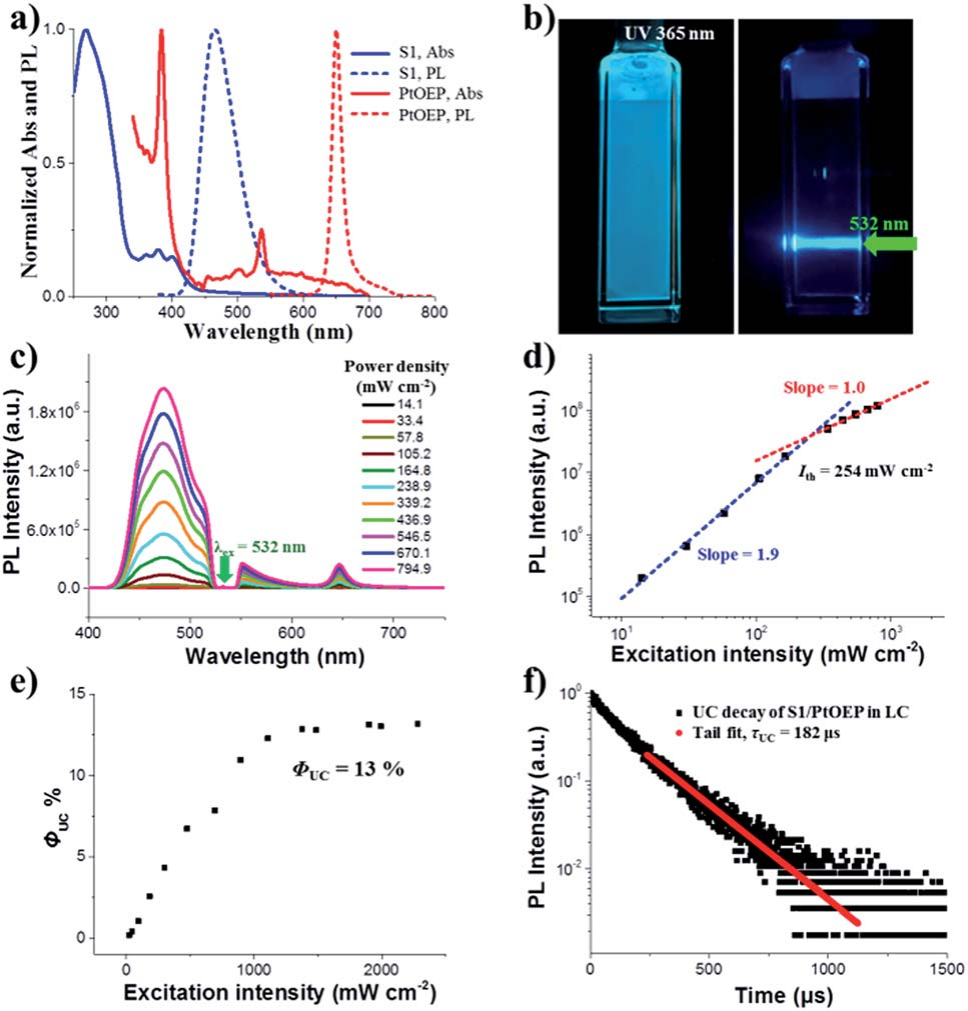
(a) Normalized absorption and emission spectra of S1 (10 wt%, *λ*_ex_ = 360 nm) and PtOEP (1 mol%, *λ*_ex_ = 532 nm) in SLC1717. (b) The left photograph of the neat film was irradiated by a 365 nm UV lamp, and the right photograph was the upconverted emission sample irradiated by a 532 nm laser (a short-pass filter was used; S1/SLC1717 = 10 wt%, PtOEP/S1 = 1 mol%). (c) Upconversion emission spectra of S1/PtOEP with different incident excitation intensities of the 532 nm laser in SLC1717. (d) Double-logarithmic plots of the UC emission intensity of S1/PtOEP in SLC1717 as a function of the excitation intensity of the laser. (e) UC quantum yield *Φ*_UC_ of S1/PtOEP in SLC1717 as a function of the excitation intensity of the laser. (f) Time-resolved upconverted emission at 470 nm of the S1/PtOEP in SLC1717 (black square), the tail fit (red circle) (*λ*_ex_ = 532 nm).

First, we fabricated a chiral nematic liquid crystal film inside a quartz cell by blending the following components: chiral emitter R(S)1, sensitizer PtOEP and room-temperature nematic liquid crystal SLC1717. Before exploring the upconverted emission properties, we tested the absorption and emission spectra of S1 and PtOEP in the liquid crystal (Fig. 2a). The S1 in SLC1717 showed the typical vibrational structure of anthracene, with its fluorescence observed with a maximum at 465 nm, which is a 20 nm red-shift compared with the spectrum in toluene solution. On the other hand, PtOEP in SLC1717 (PtOEP/S1 = 1 mol%) exhibited characteristic Soret- and Q-bands at 385 nm and 535 nm, respectively. Phosphorescence was observed at 655 nm, which is almost identical to that observed in dilute solution. Thus, PtOEP could be molecularly dispersed into the liquid crystal. Fig. 2b shows the N*LC film after irradiation using a 365 nm UV lamp and a 532 nm laser, respectively. Cyan color emission could be observed from either the fluorescence emission or the TTA-UC emission. The emission color of the fabricated neat film showed a slight red-shift from deep blue to cyan compared with the toluene solution (see the ESI, Fig. S1†). To get the best UC emission in the liquid crystal, we have carefully tested the UC emission and UC decay in various mixing weight ratio samples. It should be noted that no birefringence could be observed using a polarizing optical microscope (POM) when the weight ratio of S1/SLC1717 reached 40 wt% (see the ESI, Fig. S2†). Thus, the maximum weight ratio of S1/SLC1717, in this work, was 30 wt%. Fixing the mixing molar ratio of PtOEP/S1 at 1 mol%, by changing the weight ratio of S1/SLC1717 from 5, 10, 20 to 30 wt%, UC emission spectra with different incident power densities using the 532 nm laser were investigated (Fig. 2c and S3, ESI†). It is clear that a 10 wt% mixing ratio sample gave the maximum UC emission and the minimum residual phosphorescence from PtOEP. Double-logarithmic plots of the UC emission intensity with the weight ratio of 10 wt% are shown in Fig. 2d. A slope change from 2 to 1 was clearly observed. The blue and red lines are the fitting results with slopes of 1.9 and 1.0 in the low and high excitation intensity ranges, respectively. It involved the TTA process.^44^ The threshold excitation intensity (*I*_th_) was 254 mW cm^–2^, where TTA became the main deactivation channel for the acceptor triplet, and consequently the UC quantum yield shows saturation.^45^ A saturation quantum yield of about 13% was obtained for S1/PtOEP in SLC1717 (10 wt%) by using Rhodamine B in SLC1717 as a standard and with the theoretical maximum of 100% (Fig. 2e). These observations provide decisive evidence for the TTA-UC mechanism in the liquid crystal.

The TTA-UC mechanism could also be confirmed by the lifetime measurement. When the weight ratio of S1/PtOEP was 10 wt%, the UC emission lifetime at 470 nm was 182 μs, which is ascribed to the mechanism based on long-lived triplet species (Fig. 2f). The double-logarithmic plots and UC emission decay of other weight ratios of S1/SLC1717 are shown in Fig. S4 and S5,† respectively. In addition, after comparing the lifetime of the PtOEP in the TTA-UC process with the phosphorescence lifetime of PtOEP in SLC1717, we obtained the other evidence to prove that 10 wt% was the best weight ratio of S1/SLC1717 for UC emission (see the ESI, Fig. S6†). Subsequently, we compared the triplet–triplet energy transfer (TTET) efficiency (*Φ*_TTET_) at different weight ratios (see the ESI, Table S1†). The *Φ*_TTET_ could be calculated by evaluating the phosphorescence decay before and after adding the acceptor S1.^46^ Although the sample with a low mixing weight ratio (5 wt%) showed the highest *Φ*_TTET_ (90%), UC emission was not the best while even a higher *I*_th_ (517 mW cm^–2^) was obtained (see the ESI, Fig. S4†).

To date, the TTA-UC in a liquid crystal has rarely been reported. To carefully examine the triplet diffusion properties in this kind of room-temperature liquid crystals, we have evaluated these parameters in solution state (toluene) and liquid crystal phase. Considering the well-ordered structures in the liquid crystal phase, the triplet diffusion length should be longer than that in solution. To prove this deduction, we calculated the triplet diffusion coefficient and diffusion length in the liquid crystal (*D*_LC_, *L*_LC_) and in toluene (*D*_sol_, *L*_sol_). First, the triplet diffusion coefficient *D*_sol_ could be calculated according to the Stokes–Einstein relationship ([S1] = 1 mM; [PtOEP] = 0.01 mM),^47^1
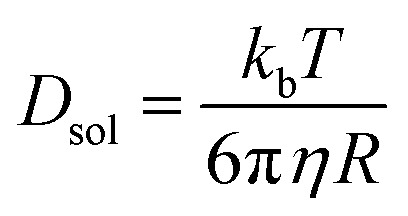
where *k*_b_ is the Boltzmann constant, *T* is the temperature (293 K), *η* is the viscosity of toluene at 293 K, and *R* is the molecular radius of the acceptor (8.5 Å). The *D*_sol_ value (in toluene solution) was calculated to be 4.3 × 10^–6^ cm^2^ s^–1^. The triplet diffusion length *L*_sol_ could be calculated using the following expression:^48^2
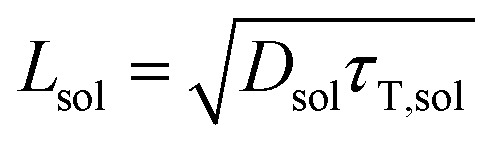
where *τ*_T,sol_ is the lifetime of an acceptor excited triplet, namely twice the value of the lifetime of the upconversion (568 μs). The value of *L*_sol_ (in toluene solution) was calculated to be 0.49 μm. The annihilation distance of the triplets (*a*_0_) could be calculated by the equation^49^3
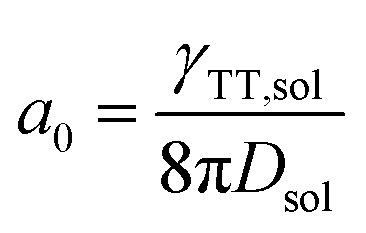
where second-order annihilation *γ*_TT,sol_ could be calculated using the following equation:^45^4
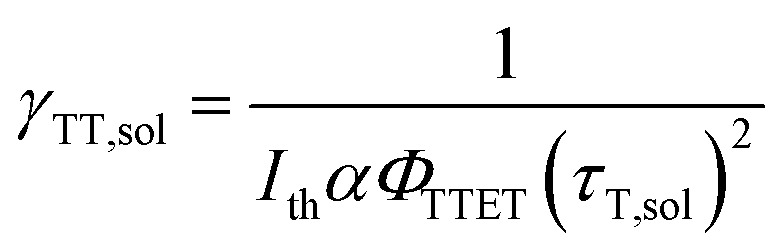



The value of *γ*_TT,sol_ was calculated to be 1.3 × 10^–11^ cm^3^ s^–1^, while the annihilation distance value *a*_0_ was calculated to be 1.2 nm. Considering that the annihilation distance for the acceptor should be a constant value whether in solution or liquid crystal, the triplet diffusion coefficient and diffusion length could be calculated by5
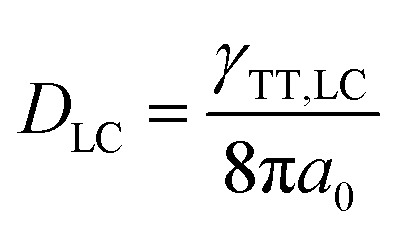



In the liquid crystal, the second-order annihilation was calculated to be *γ*_TT,LC_ = 6.6 × 10^–10^ cm^3^ s^–1^. Thus, *D*_LC_ was found to be 2.2 × 10^–4^ cm^2^ s^–1^, while the triplet diffusion length *L*_LC_ was 2.8 μm. As expected, *D*_LC_ > *D*_sol_, *L*_LC_ > *L*_sol_, that is to say, the capability of triplet diffusion in a liquid crystal is stronger, correspondingly the triplet diffusion length of the acceptor in the liquid crystal is longer than in the solution state. Moreover, the average nearest-neighbor distance between two sensitizers was calculated to be 115 nm.^50^ These results indicate that even better triplet diffusion and migration could be observed in the liquid crystal; due to the extremely high viscosity, molecular collision was severely suppressed which resulted in the relatively weak UC emission efficiency. However, the long diffusion length of the triplet was 2.8 μm in the liquid crystal, which was far enough to cover the average distance between two sensitizers (115 nm). It is expected that the capability of the triplet acceptor diffusion in the liquid crystal is stronger than in the solution state, correspondingly the triplet diffusion length of the acceptor in the liquid crystal is longer than in the solution state.

### CPL behavior in a chiral liquid crystal

**Fig. 3:**
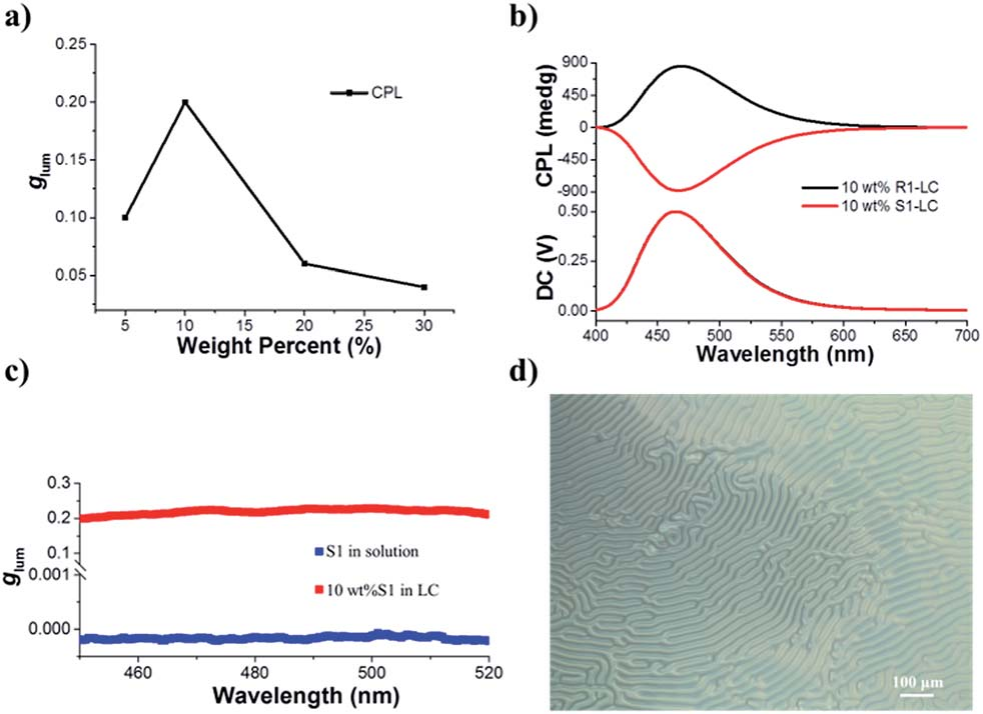
(a) The tendency of CPL dissymmetry factor *g*_lum_ with different weight ratios of S1/SLC1717 (*λ*_ex_ = 360 nm). (b) CPL spectra of 10 wt% R(S)1 in SLC1717. R1 induced SLC1717 to a right-handed N*LC, S1 induced SLC1717 to a left-handed N*LC (*λ*_ex_ = 360 nm, |*g*_lum_| = 0.2). (c) S1 in solution has the opposite *g*_lum_ value in the liquid crystal. The blue square represents the *g*_lum_ of S1 in toluene solution ([S1] = 10^–5^ M); the red square represents the *g*_lum_ of 10 wt% S1 in the liquid crystal. The values of *g*_lum_ in all emission ranges with intensity were almost the same. (d) POM image of a chiral liquid crystal. The weight ratio of S1/SLC1717 was 10 wt%.

Before we test the upconverted circularly polarized luminescence (UC-CPL) in a chiral nematic liquid crystal, it is necessary to explore the characteristics of promoted CPL in a N*LC. It has been widely demonstrated that a N*LC can work as a self-assembling one dimensional photonic crystal which could be modulated by tuning the photonic stop band.^19^,^51^–^53^ However, in this work, the stop band cannot be tuned to the visible light range because of the weak helical twisting power (HTP) of the chiral dopant. Anyhow, we have carefully checked the CPL activity of various mixing weight ratios of S1/SLC1717 in the liquid crystal cells. The tendency of the CPL dissymmetry factor *g*_lum_ at different weight ratios of S1/SLC1717 is shown in Fig. 3a. When the mixing weight ratio of S1/SLC1717 was 10 wt%, the highest *g*_lum_ value (0.2) could be observed, the corresponding mirror-image CPL and CD signals are shown in Fig. 3b and S7.†

It is already widely acknowledged that the handedness of a N*LC could be determined by the CPL signal direction in that a positive CPL signal indicates a right-handed chiral liquid crystal while a negative CPL signal means a left-handed chiral liquid crystal. Thus, it can be clearly clarified that chiral emitter R1 will induce the nematic liquid crystal to a right-handed N*LC while the addition of S1 to SLC1717 will result in a left-handed N*LC.^54^ It should be noted that the direction of the CPL signal in the liquid crystal was opposite to the one in toluene solution, which leads to the opposite value of *g*_lum_ (Fig. 3c). This could be explained as follows. The CPL activity in solution should be due to the intrinsic chiral emission property of the chiral emitter R(S)1. However, after dispersing into the nematic liquid crystal, the final CPL sign is decided by the handedness of the induced N*LC. We have also checked the polarizing optical microscope images of S1 in SLC1717 (Fig. 3d). The typical fingerprint texture proved that the N*LC had been induced successfully by the chiral emitter. In addition, we prepared the sample S1/SLC1717 with various mixing ratios from 5 to 30 wt% to measure the X-ray diffraction (XRD) (see the ESI, Fig. S8†). The results indicated that all mixing ratios showed similar diffraction patterns to those of the original nematic liquid crystal SLC1717. There was no obvious crystallization diffraction peak even at the high mixing ratio of 30 wt%, which indicated that the dopant S1 could well disperse into the liquid crystal matrix. Differential scanning calorimetry (DSC) revealed that the clearing point of SLC1717 showed a slight decline when blending with the chiral emitter (see the ESI, Fig. S9†).

### Upconverted circularly polarized luminescence in a chiral liquid crystal

**Fig. 4:**
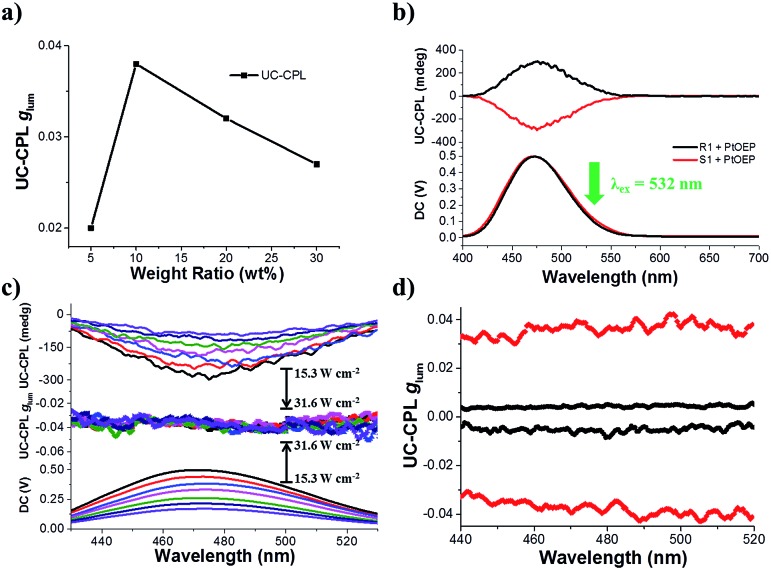
(a) The tendency of UC-CPL dissymmetry factor *g*_lum_ with different weight ratios of acceptor in the liquid crystal (PtOEP/S1 = 1 mol%, *λ*_ex_ = 532 nm). (b) UC-CPL spectra of 10 wt% R(S)1 in SLC1717 with 1 mol% PtOEP, |*g*_lum_| was nearly 0.04 (*λ*_ex_ = 532 nm). (c) UC-CPL spectra of 10 wt% S1 with different incident power densities of a 532 nm laser in a liquid crystal. The arrow indicates the spectral changes with increasing incident power density from 15.3 to 31.6 W cm^–2^. The corresponding *g*_lum_ value was almost the same as the emission value. (d) UC-CPL dissymmetry factor *g*_lum_*versus* wavelength. The black square represents R(S)1/PtOEP in toluene solution ([R(S)1] = 10^–5^ M, *λ*_ex_ = 532 nm); the red square represents R(S)1/PtOEP in SLC1717 (R(S)1/SLC1717 = 10 wt%, *λ*_ex_ = 532 nm).

The most interesting result in this work is that UC-CPL could be significantly amplified in a liquid crystal. Similarly, we have also checked the influence of different weight ratios of the acceptor in a liquid crystal on the UC-CPL. The upconverted *g*_lum_ exhibited the same tendency between promoted and upconverted CPL (Fig. 4a), while the sample with 10 wt% mixing ratio showed the highest upconverted *g*_lum_ value. As expected, a mirror-image UC-CPL signal was observed under excitation by a 532 nm green laser (Fig. 4b). It should be emphasized that the UC-CPL signal was consistent with the CPL in the liquid crystal, which indicated that the upconverted emission followed the regulation of a N*LC. With increasing the excitation intensity of the incident light, the UC-CPL increased. However, after converting to the *g*_lum_ value, the intensity was almost the same in all emission ranges (Fig. 4c). Compared with the *g*_lum_ of UC-CPL in dilute toluene solution, *g*_lum_ in the liquid crystal was one order of magnitude higher (Fig. 4d). These results indicated that whether it is promoted CPL or upconverted CPL, the optical properties of N*LC play a critical role in determining the emission properties.

Although the mechanism of photon upconversion amplified circularly polarized luminescence in dilute solution is not clear, the development of various approaches to further promote the level of upconverted circularly polarized luminescence is ongoing. The present liquid crystal system offers unprecedented opportunities for the efficient TTA-based upconversion, circularly polarized emission and upconverted circularly polarized emission. The use of room-temperature liquid crystals allows exploring the luminescence and chiroptical properties in the liquid crystal phase. The orientation of chiral acceptor molecules in the N*LC enables highly efficient triplet energy transfer and migration which might facilitate the TTA-UC. This will be a better complement to the system of triplet energy migration-based photon upconversion (TEM-UC) which has been extensively demonstrated by Kimizuka *et al.*^55^

In addition, the well-ordered chiral acceptor–acceptor arrangements would facilitate the chiral emission and upconverted chiral emission in this kind of liquid crystal phase. Thus, the integration of a chiral nematic liquid crystal amplifying CPL with TTA-based photon upconversion thus renovates the field of CPL-active functional materials and TTA-UC.

## Conclusions

In conclusion, the significance of the present study is three-fold. First, by adding the chiral acceptor molecule R(S)1 and the sensitizer PtOEP into the nematic liquid crystal, highly efficient TTA-UC could be observed in the N*LC. Moreover, efficient triplet energy diffusion and long migration length were calculated in the liquid crystal for the first time. Second, the N*LC induced by the chiral emitter exhibited good promoted CPL, while the corresponding dissymmetry factor *g*_lum_ was amplified by three orders of magnitude to 10^–1^ compared with the solution state. Third, significant amplification of UC-CPL could be observed in this N*LC system. Upconverted dissymmetry factor showed one order amplification. This work will promote the design and fabrication of chiroptical materials with not only better UC-CPL emission but also rational emission efficiency for more practical application.

## Materials and methods

### Materials

The commercial room-temperature nematic liquid crystal, SLC1717, was bought from the Chengzhi Yonghua Display Material Co., Ltd. Pt(ii)octaethyl porphyrin (PtOEP) was bought from Frontier Scientific, Inc. Rhodamine B was bought from Acros Organics.

### Instrumentation

UV-vis spectra were recorded on a Hitachi U-3900 spectrophotometer. Fluorescence spectra were measured on an F-4500 fluorescence spectrophotometer. CD and CPL spectra were measured on JASCO J-1500 and JASCO CPL-200 spectrophotometers, respectively. Lifetime measurements were recorded on the spectrometer using time-correlated single photon counting (TCSPC). Upconverted emission spectra were recorded on a Zolix Omin-λ500i monochromator with a photomultiplier tube PMTH-R 928 using an external excitation source. POM images were recorded on a Leica DM2700M upright materials microscope. Upconverted CPL spectra were recorded on a JASCO CPL-200 spectrophotometer with an external excitation source, a 532 nm semiconductor laser. DSC spectra were recorded on a PerkinElmer Diamond TG/DTA. XRD spectra were measured on a Panalytical Empyrean. Quantum yield was measured on an Edinburg FLS-980 fluorescence spectrometer with a calibrated integrating sphere.

### Characterization and methods

The sample used to investigate the UV-vis and fluorescence spectra, lifetime, upconversion emission and UC-CPL was fabricated by the following method. Firstly, 1 mg S1 and 9 mg SLC1717 were added into a 1 mL centrifuge tube, and then mixed with 1 mL toluene. After that, the resulting solution was sonicated for about 1 min to obtain a good solution. Finally, 400 μL of the solution was transferred into a quartz cell and toluene was evaporated slowly using a vacuum pump. The thin chiral nematic liquid crystal film was generated in the inner face of the quartz cell. It should be noted that evaporation of toluene should be carried out at an appropriate velocity, otherwise the generated chiral nematic liquid crystal will not be uniform. The chiral nematic liquid crystal containing the PtOEP was fabricated in the same way, 10.6 μL PtOEP (10^–3^ M) should be added with 1 mg S1. The UV-vis spectra were recorded from 200 nm to 800 nm. Emission spectra were recorded with an excitation wavelength of 360 nm. The samples were excited with an incidence angle of 45° to the quartz cell surface and the fluorescence was detected along the normal. Time-resolved upconverted emission spectra were measured with an excitation wavelength of 532 nm laser and detected at 470 nm. The upconverted lifetimes have been fitted with a single exponential decay. Spectra of UC emission and UC-CPL were measured with the excitation wavelength of a 532 nm laser. The semiconductor laser used in our experiment was bought from Changchun New Industries Optoelectronics Technology Co., Ltd. The bandwidth was < 0.2 nm, Gaussian beam in spatial mode TEM00, with a polarization ratio of 1 : 100, the polarization direction being horizontal. The sample used for CPL spectra was fabricated by the following method. Firstly, 2 mg S1 and 18 mg SLC1717 were dissolved in 1 mL toluene. After drying the toluene, N*LC was added into the liquid crystal cell. CPL spectra were recorded with the excitation wavelength of a 360 nm Xe-lamp. The sample used for CD spectra and POM measurement was prepared by the following method. 1 mg S1 and 9 mg SLC1717 were dissolved in 1 mL toluene. Subsequently, the resulting solution was sonicated for about 1 min to obtain a solution. 50 μL of the solution was transferred to a 0.1 mm quartz cell and the solvent was evaporated. CD spectra were recorded from 200 nm to 800 nm. By casting the solution on a quartz plate, uniform film could be formed after evaporating the solvent. The film sample could be used for POM measurement and XRD measurement.

### Determination of TTA-UC quantum yield by a relative method

The upconverted emission quantum efficiency was determined relative to a standard according to the following equation:
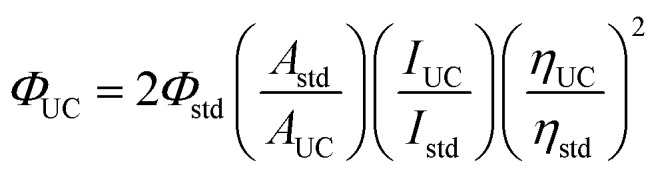
where *Φ*, *A*, *I* and *η* represent the quantum yield, intensity of absorbance at 532 nm, integrated photoluminescence spectral profile, and refractive index of the solvents used as a standard, respectively. The subscripts UC and std denote the parameters of the upconversion and standard systems. Since the standard and the UC dyes are doped in the same liquid crystal, the refractive indices are the same. Therefore, under our experimental conditions, the upconverted emission quantum efficiency *Φ*_UC_ could be calculated using the equation. For the UC system, the weight ratio of S1/SLC1717 was 10 wt%, the molar ratio of PtOEP/S1 was 1%. For the standard system, the concentration of Rhodamine B was the same as the concentration of PtOEP. The UC system was tested from 400 nm to 530 nm, and the standard system was tested from 540 nm to 750 nm. The UC quantum yield was determined relative to a standard, Rhodamine B in SLC1717 (*Φ*_std_ = 0.441) under 532 nm excitation. The result of upconverted emission quantum efficiency was the mean value for parallel testing three times.

## Conflicts of interest

There are no conflicts to declare.

## Supplementary Material

Supplementary information
