# Supplementary material for: Photon-upconverting chiral liquid crystal: significantly amplified upconverted circularly polarized luminescence
Source: Chem Sci. 2018 Oct 2;10(1):172–8. doi: 10.1039/c8sc03806f (PMC6330689; doi:10.1039/c8sc03806f)
Supplement: Supplementary file 1 [file SC-010-C8SC03806F-s001.pdf]

Electronic Supplementary Material (ESI) for Chemical Science.
This journal is © The Royal Society of Chemistry 2018

Electronic supplementary information

Photon-upconverting chiral liquid crystal: significantly amplified upconverted circularly polarized luminescence

Xuefeng Yang,[ab] Jianlei Han,[b] Yafei Wang,[c] and Pengfei Duan*[bd]

[a] College of Chemistry, Key Lab of Environment-Friendly Chemistry and Application of the Ministry of Education, Xiangtan University, Xiangtan 411105, P.R. China.
[b] CAS Center for Excellence in Nanoscience, CAS Key Laboratory of Nanosystem and Hierarchical Fabrication, Division of Nanophotonics, National Center for Nanoscience and Technology (NCNST), No. 11 ZhongGuanCun BeiYiTiao, Beijing 100190, P. R. China E-mail: duanpf@nanoctr.cn.
[c] Science and Engineering, Jiangsu Collaboration Innovation Center of Photovoltaic Science and Engineering, Changzhou University, Changzhou 213164, P. R. China.
[d] University of Chinese Academy of Sciences, Beijing 10049, P. R. China.

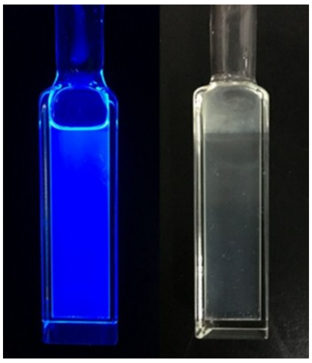

Fig. S1 Left quartz cell was the toluene solution of S1, PtOEP and SLC1717 under the 365 nm ultraviolet lamp, the emission light was deep blue. Right quartz cell showed the neat film after drying the toluene.

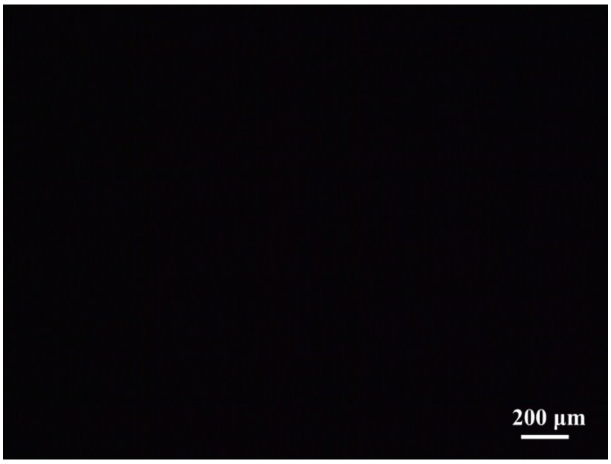

Fig. S2 POM image of the weight ratio of S1/SLC1717 was 40 wt%. Birefringence could not be observed entirely.

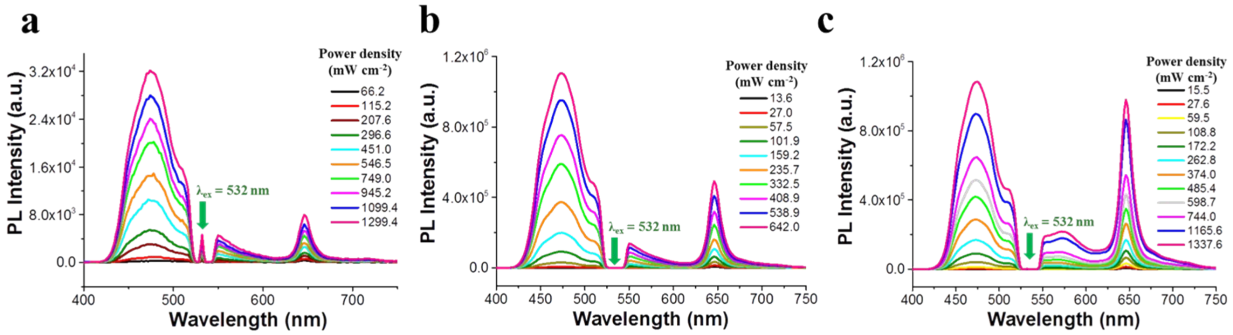

Fig. S3 Upconversion emission spectra of different weight ratios of S1/PtOEP in LC with different incident power density of 532 nm laser: a) S1/SLC1717 = 5 wt%, PtOEP/S1 = 1 mol%. b) S1/SLC1717 = 20 wt%, PtOEP/S1 = 1 mol%. c) S1/SLC1717 = 30 wt%, PtOEP/S1 = 1 mol%.

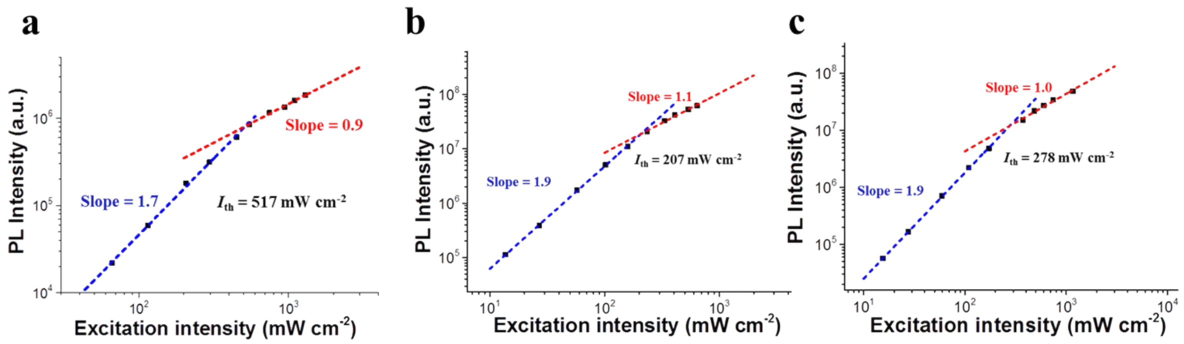

Fig. S4 Double-logarithmic plots of the UC emission intensity of different weight ratios of S1/PtOEP in LC: a) S1/SLC1717 at 5 wt%, PtOEP/S1 = 1 mol%. b) S1/SLC1717 = 20 wt%, PtOEP/S1 = 1 mol%. c) S1/SLC1717 = 30 wt%, PtOEP/S1 = 1 mol%.

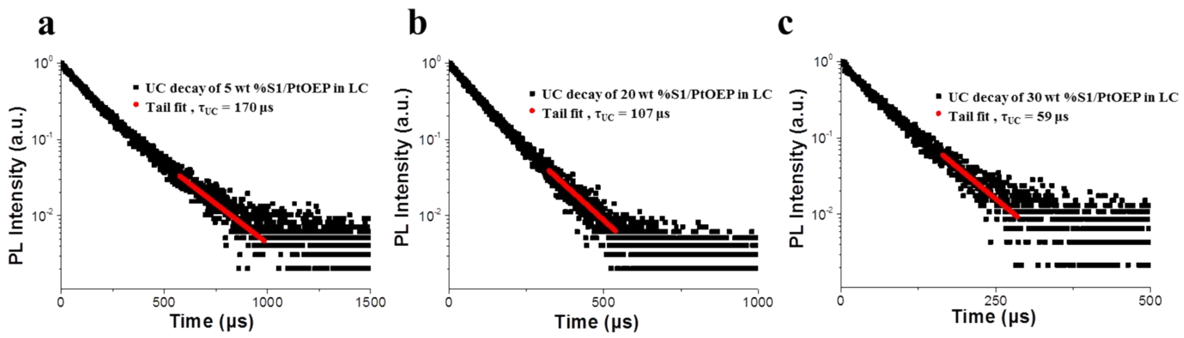

Fig. S5 Time resolved upconverted emission at 470 nm of the different weight ratios of S1/PtOEP in LC: a) S1/SLC1717 = 5 wt%, PtOEP/S1 = 1 mol%. b) S1/SLC1717 = 20 wt%, PtOEP/S1 = 1 mol%. c) S1/SLC1717 at 30 wt%, PtOEP/S1 = 1 mol%.

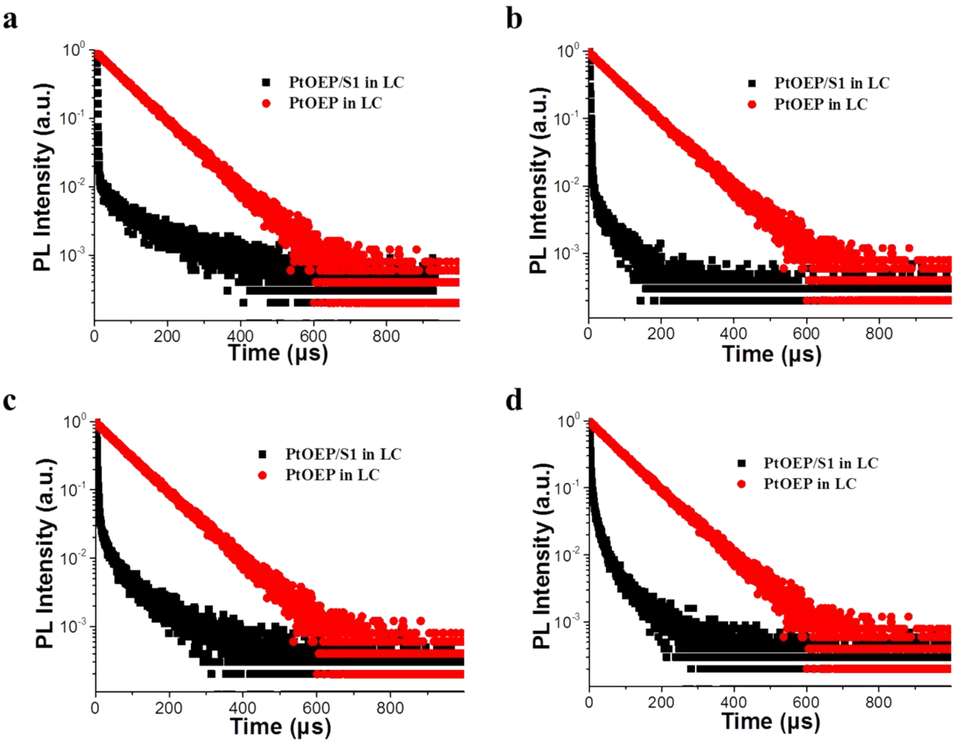

Fig. S6 Lifetime of the PtOEP at 646 nm in the mixture of S1/PtOEP in LC (black square), lifetime of the PtOEP in SLC1717 at 646 nm (red circle): a) S1/SLC1717 = 5 wt%, PtOEP/S1 = 1 mol%. b) S1/SLC1717 = 10 wt%, PtOEP/S1 = 1 mol%. c) S1/SLC1717 = 20 wt%, PtOEP/S1 = 1 mol%. d) S1/SLC1717 at 30 wt%, PtOEP/S1 = 1 mol%.

Table S1. UC decay and phosphorescence decay of PtOEP with or without S1 in liquid crystal.

wt %	τ_{UC} (μs)	τ_D (μs)	τ_1 (μs)	τ_2 (μs)	τ_1 %	Φ_{TTET}
5 %	170	83	53	2	94	90 %
10 %	182	86	67	8	90	79 %
20 %	107	30	67	13	81	66 %
30 %	59	27	34	8	73	57 %

UC emission lifetime of S1/PtOEP (τ_{UC}) at 470 nm showed single exponential. τ_D was lifetime of PtOEP in LC at 646 nm. Lifetimes of τ_1 and τ_2 were the decay of the residual PtOEP phosphorescence at 646 nm showed clearly biexponetial. Φ_{TTET} represents the transfer efficiency.

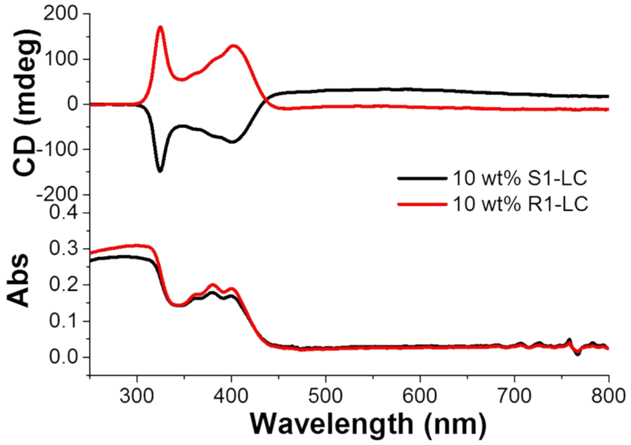

Fig. S7 CD spectra of R(S)1 in SLC1717 showed mirror-image signals. The weight ratio of R(S)1/SLC1717 was 10 wt%.

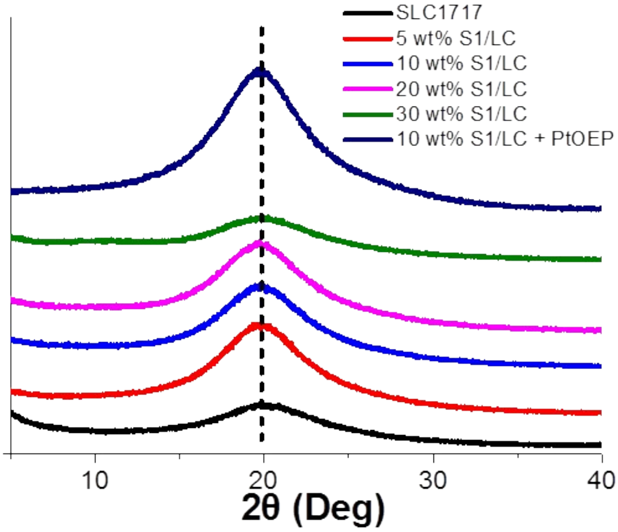

Fig. S8 XRD showed that SLC1717 was the non-crystal structure while adding S1 into the SLC1717 did not influence the structure of liquid crystal. Moreover, the structure of liquid crystal did not change after mixing with PtOEP.

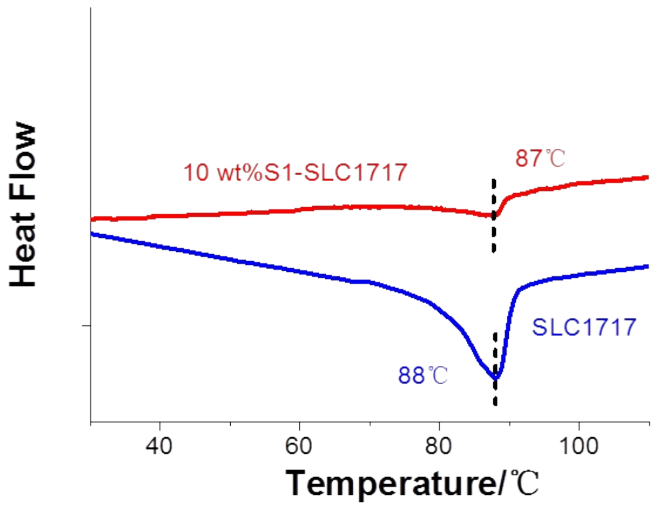

Fig. S9 DSC cooling trace of SLC1717 and SLC1717 blending with 10 wt% S1. Clearing point showed a slight decline.
